# Health information literacy mediates the relationship between active information seeking and HPV-related knowledge among female undergraduates: the moderating role of information channel characteristics

**DOI:** 10.3389/fpubh.2026.1947194

**Published:** 2026-10-01

**Authors:** Min Xie, Miao Zhao

**Affiliations:** 1 Department of Cosmetic Plastic and Burn Surgery, The First Affiliated Hospital of Chengdu Medical College, Chengdu, Sichuan, China; 2 College of Pharmacy, Chengdu Medical College, Chengdu, Sichuan, China

**Keywords:** health information literacy, human papillomavirus, information channel characteristics, information seeking behavior, knowledge, moderated mediation model

## Abstract

**Background:**

Cervical cancer remains a significant global public health challenge. Although human papillomavirus (HPV) vaccination effectively reduces incidence, coverage among Chinese female undergraduates remains suboptimal. Adequate HPV-related knowledge (HK) is essential for promoting vaccination uptake; however, the mechanism by which HPV-related active information seeking behavior (AISB) translates into accurate HK through health information literacy (HIL) remains underexplored. This study examines the mediating effect of HIL in the AISB–HK relationship and the moderating roles of primary information access channel type (CT) and channel trust preference (CTR), providing empirical evidence for targeted university-based HPV health education.

**Methods:**

A cross-sectional survey was conducted from May to June 2024 among 519 female undergraduates at a comprehensive university in China. Participants completed a structured questionnaire assessing demographic characteristics, AISB, HIL, HK, CT, and CTR. Mediation and moderated mediation analyses were performed using the PROCESS macro (Model 4 and Model 14), controlling for demographic covariates.

**Results:**

HIL partially accounted for the association between AISB and HK (indirect effect = 0.056, 95% CI [0.019, 0.093]). Both CT and CTR significantly moderated the HIL–HK pathway. The HIL–HK association was significant only among participants relying on non-professional channels (*B* = 0.112, *p* < 0.001; professional channels: *B* = 0.018, *p* = 0.521; index of moderated mediation = −0.075, 95% CI [−0.142, −0.011]). For CTR, the association was significant in both groups but approximately four times stronger among participants trusting non-professional channels (*B* = 0.210, *p* < 0.001) than among those trusting professional channels (*B* = 0.049, *p* = 0.019; index of moderated mediation = −0.128, 95% CI [−0.235, −0.046]).

**Conclusion:**

HIL was a significant intermediate link in the association between AISB and HK, and information channel characteristics were associated with the strength of this association. Targeted interventions aimed at enhancing HIL and optimizing health communication channels may help improve HK among college students and advance cervical cancer prevention.

## 1 Introduction

Cervical cancer, predominantly caused by persistent infection with high-risk human papillomavirus (HPV) (1), remains a major global public health challenge. It ranked fourth in both incidence and mortality among women globally in 2022, with approximately 660,000 new cases and 350,000 deaths reported (2). The advent of HPV vaccines has brought hope for the prevention and even elimination of cervical cancer. Many studies have consistently demonstrated the efficacy of HPV vaccination in preventing HPV infection and significantly lowering cervical cancer incidence (3, 4). However, HPV vaccination coverage in China remains relatively low. Data indicate that by 2022, the coverage rate for the first dose of the HPV vaccine among Chinese women aged 9–45 was only 10.15%, with the full-series completion rate standing at merely 6.21% (5).

Adequate HPV-related knowledge (HK) is a critical prerequisite for improving vaccination intention and coverage rates (6–8). In China, female university students constitute a population at high risk of HPV exposure and represent a primary target for HPV vaccination (9, 10). However, this group consistently exhibits low levels of HK and suboptimal vaccination rates (8, 11). In the current digital era, social media and online platforms have become predominant sources for health information acquisition among students (12, 13). While the internet enhances accessibility to such information, it simultaneously exposes students to a complex information environment where multiple channels coexist with significant variations in content quality (14, 15). This environment may profoundly influence health information processing, knowledge accumulation, and responses to health issues (16). Therefore, investigating the behaviors, channels, and determinants of HK acquisition among female university students holds significant practical value for advancing cervical cancer prevention and control efforts.

Previous research on determinants of health knowledge has identified key factors and their associated findings:

- Health information seeking behavior. Active engagement in health information seeking is significantly positively associated with health-related knowledge levels (17–21).
- Health literacy (HL). Defined as “the degree to which individuals have the capacity to obtain, process, and understand basic health information and services needed to make appropriate health decisions” (22), HL demonstrates a significant positive correlation with health-related knowledge levels (23–25).
- Information literacy (IL). Referring to “the ability to recognize when information is needed and to locate, evaluate, and use effectively the needed information” (26), studies examining IL’s impact on health-related knowledge primarily focus on healthcare professionals. Evidence indicates that IL is positively correlated with certain domains of professional knowledge and competencies among this population (27, 28), while little is known about its role in general populations.
- Information source. Greater source diversity correlates positively with health knowledge quality (29), and individuals using professional channels exhibit higher knowledge levels than those relying on non-professional sources (21).

Existing research has predominantly focused on the direct association between single influencing factors and health knowledge, while the underlying mechanisms from information exposure to systematic knowledge acquisition remain largely unexplored. This research gap is particularly pronounced in the HPV domain: only a limited number of studies have examined the correlation between HL and HK (30–32). Furthermore, an important variable—health information literacy (HIL)—has rarely been incorporated into studies on determinants of health knowledge. HIL has been defined as “the set of abilities needed to recognize a health information need, identify likely information sources and use them to retrieve relevant information, assess the quality of the information and its applicability to a specific situation, and analyze, understand, and use the information to make good health decisions” (33). As a specific application of information literacy in the health domain, HIL also constitutes an essential component of health literacy (34). In the complex information environment of the digital age, HIL serves as a more focused construct that functions as a “safety barrier” for filtering and evaluating health information (34), holding significant value for individual health.

To address the aforementioned research gaps, the present study proposes a theoretical framework in which HIL serves as a mediator in the relationship between HPV-related active information seeking behavior (AISB) and HPV-related knowledge (HK), with this indirect pathway further moderated by information channel characteristics—namely, primary information access channel type (CT) and channel trust preference (CTR). This conceptual model was examined among Chinese female undergraduates. Based on this framework and the empirical foundations outlined above, three hypotheses were proposed:

*H1*: HIL mediates the positive relationship between AISB and HK.

*H2*: CT moderates the indirect effect, such that the HIL–HK pathway is stronger among participants who primarily use non-professional channels than among those who use professional channels.

*H3*: CTR moderates the indirect effect, such that the HIL–HK pathway is stronger among participants who trust non-professional channels than among those who trust professional channels.

The conceptual model is presented in Figure 1.

**Figure 1:**
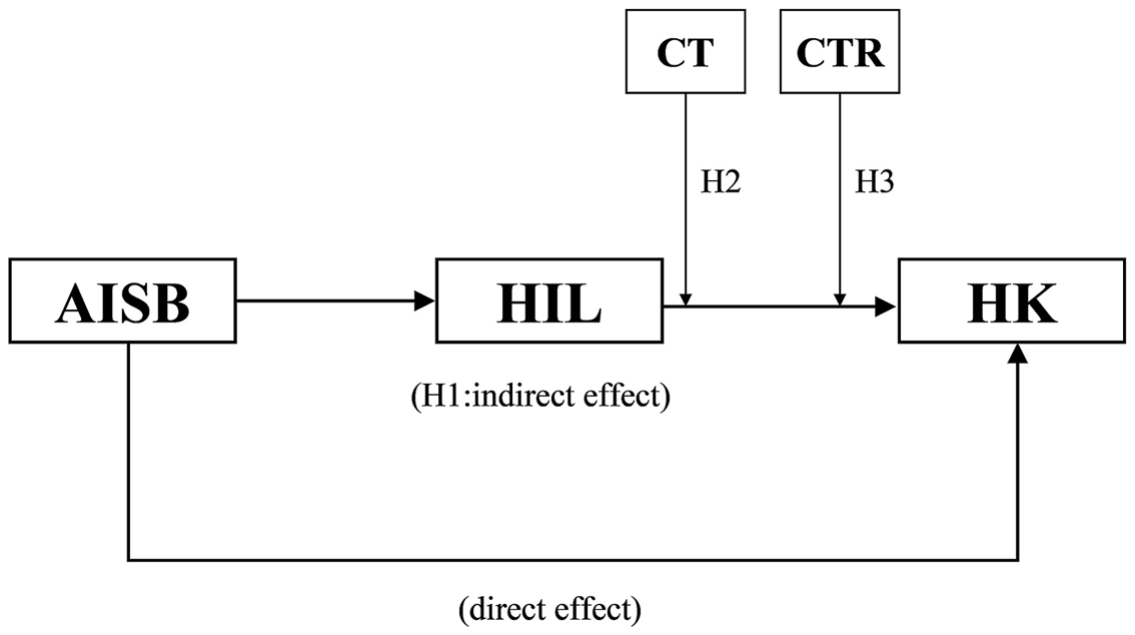
Conceptual moderated mediation model. H1 represents the indirect effect of AISB on HK through HIL. H2 and H3 represent the moderating effects of CT and CTR on the HIL → HK pathway, respectively. The direct effect of AISB on HK is also estimated.

By systematically elucidating this mediating mechanism, the study aims to deepen understanding of the pathway through which health information is translated into knowledge among young adults, and to provide empirical evidence for optimizing targeted HPV-related health education strategies in university settings.

## 2 Materials and methods

### 2.1 Study design

A cross-sectional design was employed for this study, which took place between May and June 2024 at a comprehensive university in Chengdu, China. Participants were recruited from the population of full-time female undergraduates at the university. Data were gathered through a self-administered electronic questionnaire designed and distributed via Wenjuanxing,^1^ a prevalent online survey platform in China. During the design of the online questionnaire, we enforced mandatory responses: respondents could not submit the questionnaire unless all items were completed. Therefore, there were no missing values for any item across all returned questionnaires. Eligible participants were invited to complete the survey by scanning a quick response (QR) code. The reporting of this study strictly followed the Strengthening the Reporting of Observational Studies in Epidemiology (STROBE) guidelines.

### 2.2 Ethical considerations

The study was conducted in accordance with the guidelines of the Declaration of Helsinki and approved by the Institutional Review Board (IRB) of The First Affiliated Hospital of Chengdu Medical College, China. According to Item 2, Article 32 of the “Ethical Review Measures for Life Sciences and Medical Research Involving Humans” issued by the People’s Republic of China, studies utilizing anonymized data that pose no harm to human subjects, do not involve sensitive personal information, and are free from commercial interests may be exempted from full ethical review. Consequently, given the purely anonymous nature of this survey, formal ethical review was deemed unnecessary. On this basis, the IRB approved the study via fast-track review process without an approval number. Electronic informed consent was obtained from all participants. All collected data were treated with strict confidentiality and were used solely for research purposes.

### 2.3 Sampling and participants

A stratified cluster sampling method was employed to recruit eligible participants from the full-time female undergraduate population at the university. Stratification was based on two dimensions: academic major and academic year. Specifically, four academic majors were selected, spanning two disciplinary fields: science and engineering (Computer Science and Civil Engineering) and humanities and social sciences (Law and Business Administration). Students from the first, second, and third academic years were included. Because stratification was conducted at the level of individual academic majors rather than disciplinary fields, this yielded a total of 12 strata (4 majors × 3 academic years). Within each stratum, two intact classes were randomly selected. All female students in the selected classes were invited to participate in the survey.

Sample size estimation was performed according to existing literature, and the corresponding formula is as follows:

$$n=\frac{Z_{\alpha /2}^{2}\times p(1-p)}{d^{2}}$$

In the formula, the parameter *Z*_*α/*2_ represents the *Z*-score corresponding to a two-sided 95% confidence level (*α* = 0.05), which was set to 1.96. The parameter *d* represents the margin of error. In this study, *d* was set to 0.05 for the calculation. The parameter *p* represents the estimated proportion of female undergraduate students with awareness of HK. Based on recent studies (35–37), the awareness rates of HPV and the HPV vaccine among female college students in China range from 59.9 to 93.2% and 57.3 to 87.5%, respectively. Therefore, we set the expected awareness rate of HK at 70%, and *p* was assigned a value of 0.7 for the calculation. Based on the above parameters, the initial calculation yielded a minimum valid sample size of 323 participants. To compensate for potential invalid responses and missing data, the target sample size was inflated by 15%, resulting in a final required minimum sample size of 372 participants.

Eligible participants were: (a) full-time Chinese female undergraduate students in their first, second, or third academic year; (b) individuals who had heard of HPV and the HPV vaccine; and (c) those who provided voluntary informed consent. Participants were excluded if they: (a) had received at least one dose of the HPV vaccine; (b) had contraindications or medical conditions precluding vaccination; (c) self-reported cognitive impairments that could compromise questionnaire understanding; or (d) refused to participate in the study.

### 2.4 Variables and measurements

#### 2.4.1 Demographic characteristics

The following demographic characteristics were assessed in this survey: age, academic major, academic year, residence (urban or rural), monthly living expense (China Yuan, CNY), and sexual experience (yes or no).

#### 2.4.2 HPV-related active information seeking behavior

AISB was measured using a self-developed 3-item brief scale. The three items were: (a) “I actively pay attention to HPV-related information around me”; (b) “I actively search for or consult about HPV-related information”; (c) “I actively discuss HPV-related topics with others,” corresponding to three behavioral facets: active attention, active search and consultation, and active interpersonal discussion. The scale was scored on a 5-point Likert scale from 1 (strongly disagree) to 5 (strongly agree). A total score was derived by summing all item responses, with higher scores indicating a higher level of HPV-related active information seeking behavior. Prior to the formal survey, a pilot study was conducted to evaluate the clarity and reliability of the self-developed AISB scale. The pilot sample size was determined according to the rule of thumb of 5–10 participants per scale item. As the AISB scale contained three items, the recommended sample size was 15–30. A convenience sample of 30 female undergraduates was recruited. The pilot results indicated acceptable internal consistency (Cronbach’s *α* = 0.72), and minor wording refinements were made based on participant feedback before the scale was administered in the formal survey. In the formal study, the Cronbach’s *α* coefficient of the scale was 0.80, indicating good internal consistency.

#### 2.4.3 Health information literacy

HIL was measured using the Chinese revised version of the Screening Tool for Assessing Everyday Health Information Literacy (EHIL), originally developed by Niemelä et al. (38) as a 10-item instrument and later translated and revised by Luo et al. (39). The Chinese version retained the original 10 items and added 4 new items, yielding a 14-item scale. Each item is rated on a 5-point Likert scale (1 = strongly disagree, 5 = strongly agree). Items 1, 2, 3, 6, 7, 9, 12, 13, and 14 are positively worded, whereas items 4, 5, 8, 10, and 11 are reverse-coded. After reverse-coding, the theoretical total score ranges from 14 to 70, with higher scores indicating greater HIL. In the current study, Cronbach’s α for the scale was 0.67, which is close to the value of 0.70 reported in the original Chinese validation (39). This level of internal consistency is considered acceptable for exploratory research and in studies with relatively homogeneous samples (40).

#### 2.4.4 HPV-related knowledge

HK was measured using a self-developed HPV-Related Knowledge Questionnaire, which comprises two subdomains: HPV knowledge and HPV vaccine knowledge. The HPV knowledge subdomain includes seven single-choice items and one multiple-response item, while the HPV vaccine knowledge subdomain consists of four single-choice items and one multiple-response item.

Each single-choice item provides four response options, including an “I don’t know” option. One point was awarded for selecting the correct answer, and 0 points were given for an incorrect response or selection of the “I do not know” option. Each multiple-response item contains five response options (including an “I do not know” option) with multiple correct answers. A full score of 1 point was granted only when all correct options were selected; any incorrect choice, omission of correct options, or selection of the “I do not know” option resulted in a score of 0. The total scores of the questionnaire range from 0 to 13, with higher scores indicating a higher level of HK.

Content validity was evaluated by a panel of six experts in epidemiology and pharmacy. Regarding content validity, the item-level content validity index (I-CVI) of the questionnaire ranged from 0.83 to 1.00, and the scale-level content validity index based on universal agreement (S-CVI/UA) was 0.85. The questionnaire was included in the pilot study, in which no major ambiguities were identified. The Kuder–Richardson formula 20 (KR-20) coefficient for the questionnaire in the formal study was 0.71, indicating acceptable internal consistency. The distribution of HK scores was approximately symmetric (skewness = −0.791, kurtosis = 0.053).

#### 2.4.5 HPV-related information channel characteristics

The measurement of HPV-related information channel characteristics was based on a review of commonly used health information sources among university students, which included two dimensions: CT and CTR. All channels were categorized into two groups: professional medical and health channels, and non-professional medical and health channels, covering a total of eight specific information sources.

Professional medical and health channels included: (a) professional medical websites or applications (e.g., DXY.cn, Chunyu Doctor); (b) health education and communication from medical staff (e.g., health lectures, one-to-one consultation); (c) promotional materials released by medical and health organizations (e.g., HPV brochures, bulletin boards in schools and communities).

Non-professional medical and health channels included: (a) traditional media (e.g., television, radio, newspapers); (b) general information websites or apps (e.g., Sina, Toutiao); (c) social media platforms (e.g., WeChat, TikTok, Xiaohongshu); (d) communication with family, friends and classmates; (e) other sources.

For the primary access channel type, participants were asked to select the principal channel through which they had obtained HPV-related information. They were then classified into the professional channel group or the non-professional channel group based on the category of their selected primary channel (the “other sources “option was classified into the non-professional group).

For channel trust preference, participants selected the single most trusted channel for HPV information. Similarly, they were classified into the professional-trust group or the non-professional-trust group based on the category of their most trusted channel (the “other sources “option was classified into the non-professional-trust group).

### 2.5 Data collection

Data collection was conducted during class breaks without financial compensation. The procedure involved: (a) male students were asked to leave the classroom; (b) eligible female students were briefed on the study and instructed on questionnaire completion; (c) the anonymous survey was distributed via QR code, with an IP restriction applied to prevent duplicate submissions; and (d) respondents completed and submitted the questionnaire voluntarily. Invalid responses were excluded based on inconsistent demographic data or uniform answer patterns. Of the 590 questionnaires collected, 71 were discarded, yielding 519 valid responses with an overall valid response rate of 87.97%.

### 2.6 Statistical analysis

All statistical analyses were conducted using SPSS 23.0 (IBM Corp., Armonk, NY, United States) with the PROCESS macro Version 4.3 (41). Descriptive statistics were computed to characterize the study sample. Categorical variables were presented as frequencies (n) and percentages (%). Continuous variables were summarized as means with standard deviations (SD). Group differences in HIL, HK, and AISB scores were examined using parametric tests. Independent samples *t*-tests were applied for between-group comparisons across dichotomous demographic variables. One-way analysis of variance (ANOVA) was used to compare scores across multi-level demographic variables. When the homogeneity of variances assumption was met (Levene’s test *p* > 0.05), Tukey’s HSD test was used for post-hoc comparisons; otherwise, the Games-Howell test was applied.

Pearson correlation analysis was performed to examine the bivariate associations among HIL, AISB, and HK. Mediation and moderated mediation analyses were conducted using the PROCESS macro (Model 4 and Model 14, respectively). The mediation hypothesis (H1) was tested using Model 4, with AISB as the independent variable, HK as the dependent variable, and HIL as the mediator. The moderated mediation hypotheses (H2 and H3) were tested using two separate Model 14 analyses, which test moderation of the second-stage path, with CT and CTR entered as moderators, respectively. All models controlled for age, academic major, academic year, residence, monthly living expenses, and sexual experience.

Indirect effects and indices of moderated mediation were evaluated using 95% bias-corrected bootstrap confidence intervals based on 5,000 resamples. An effect was considered statistically significant when the 95% bootstrap confidence interval did not include zero. A significance level of *α* = 0.05 (two-tailed) was adopted for all hypothesis tests.

## 3 Results

### 3.1 Participants’ demographic characteristics

The participants were predominantly aged 20–21 years (51.6%, *n* = 268) and were in their second year of university (37.2%, *n* = 193). Among the four majors, Law was the most represented (27.7%, *n* = 144). The majority of the participants came from rural areas (68.4%, *n* = 355) and had monthly living expense ranging from 1,001 to 1,500 CNY (61.5%, *n* = 319). Furthermore, the vast majority of the participants reported no prior sexual experience (87.5%, *n* = 454). Detailed information is provided in Table 1.

**Table 1:** Demographic characteristics of participants (*n* = 519).

Variable	Group	Frequency (*n*)	Percent (%)
Age	① ≤19	194	37.4
	② 20 ~ 21	268	51.6
	③ ≥22	57	11.0
Academic major	①Computer Science	137	26.4
	②Civil Engineering	114	22.0
	③Law	144	27.7
	④Business Administration	124	23.9
Academic year	① 1st	187	36.0
	② 2nd	193	37.2
	③ 3rd	139	26.8
Residence	① Urban	164	31.6
	② Rural	355	68.4
Monthly living expenses (CNY)	① ≤1,000	33	6.4
	② 1,001 ~ 1,500	319	61.5
	③ 1,501 ~ 2000	125	24.1
	④ ≥2001	42	8.1
Sexual experience	① Yes	65	12.5
	② No	454	87.5

### 3.2 Differences in AISB by demographic characteristics

In this study, the mean score for AISB among the participants was 9.92 ± 2.10 (out of a maximum of 15 points), corresponding to a score rate of 66.1%, which indicates a moderate level of HPV-related active information seeking behavior in this population.

Statistically significant differences in AISB scores were only detected across monthly living expense groups (*p* = 0.002, *η^2^* = 0.027). No significant between-group differences in AISB scores were found for age, academic majors, academic years, residence, or sexual experience. Detailed information is provided in Table 2.

**Table 2:** Comparison of AISB scores across demographic characteristics (M ± SD).

Variable	Group	AISB
Age	① ≤19	9.76 ± 2.09
	② 20 ~ 21	10.10 ± 2.11
	③ ≥22	9.65 ± 2.10
	*F*	1.970
	*p*	0.140
Academic major	① Computer Science	9.98 ± 1.87
	② Civil Engineering	9.73 ± 2.36
	③ Law	10.22 ± 2.15
	④ Business Administration	9.70 ± 2.02
	*F*	1.751
	*p*	0.156
Academic year	① 1^st^ year	9.76 ± 2.12
	② 2^nd^ year	10.04 ± 2.04
	③ 3^rd^ year	9.98 ± 2.17
	*F*	0.920
	*p*	0.399
Residence	① Urban	9.98 ± 2.16
	② Rural	9.90 ± 2.08
	*t*	0.388
	*p*	0.698
Monthly living expenses (CNY)	① ≤1,000 CNY	8.97 ± 1.93
	② 1,001 ~ 1,500 CNY	9.81 ± 2.02
	③ 1,501 ~ 2000 CNY	10.34 ± 2.23
	④ >2000 CNY	10.31 ± 2.19
	*F*	4.840
	*p*	0.002
	*η^2^*	0.027
	Tukey HSD *post hoc* test	① < ③, ④
Sexual experience	① Yes	10.17 ± 2.19
	② No	9.89 ± 2.09
	*t*	1.010
	*p*	0.313

### 3.3 Differences in HIL by demographic characteristics

In this study, the observed total scores ranged from 28 to 70, and the mean score for HIL among the participants was 47.23 ± 5.37 (out of a maximum of 70 points), corresponding to a score rate of 67.5%, which indicates a moderate-to-high level of HIL in this population.

Statistically significant differences in HIL scores were only detected across monthly living expense groups (*p* = 0.017, *η^2^* = 0.020). No significant between-group differences in HIL scores were found for age, academic majors, academic years, residence, or sexual experience. Detailed information is provided in Table 3.

**Table 3:** Comparison of HIL scores across demographic characteristics (M ± SD).

Variable	Group	HIL
Age	① ≤19	47.85 ± 5.481
	② 20 ~ 21	46.92 ± 5.402
	③ ≥ 22	46.61 ± 4.705
	*F*	2.109
	*p*	0.122
Academic major	① Computer Science	47.55 ± 4.75
	② Civil Engineering	46.96 ± 5.40
	③ Law	46.67 ± 5.73
	④ Business Administration	47.78 ± 5.54
	*F*	1.218
	*p*	0.302
Academic year	① 1^st^ year	47.88 ± 5.56
	② 2^nd^ year	46.69 ± 5.08
	③ 3^rd^ year	47.10 ± 5.46
	*F*	2.392
	*p*	0.093
Residence	① Urban	47.66 ± 5.28
	② Rural	47.03 ± 5.41
	*t*	1.250
	*p*	0.212
Monthly living expenses (CNY)	① ≤1,000 CNY	45.97 ± 5.22
	② 1,001 ~ 1,500 CNY	46.88 ± 4.96
	③ 1,501 ~ 2000 CNY	48.50 ± 6.08
	④ >2000 CNY	47.12 ± 5.77
	*F*	3.422
	*p*	0.017
	*η^2^*	0.020
	Tukey HSD post hoc test	② < ③
Sexual experience	① Yes	46.91 ± 5.69
	② No	47.28 ± 5.33
	*t*	−0.519
	*p*	0.604

### 3.4 Differences in HK by demographic characteristics

In this study, the mean score for HK among the participants was 8.00 ± 2.70 (out of a maximum of 13 points), corresponding to a score rate of 61.5%, which indicates a moderate level of HK in this population.

There were statistically significant differences in HK scores across groups based on age (*p* = 0.001, *η^2^* = 0.028), academic year (*p* < 0.001, *η^2^* = 0.032), monthly living expense (*p* = 0.036, *η^2^* = 0.017), and sexual experience (*p* = 0.004, Cohen’s *d* = 0.397). No significant between-group differences in HK scores were found for academic majors or residence. Detailed information is provided in Table 4.

**Table 4:** Comparison of HK scores across demographic characteristics (M ± SD).

Variable	Group	HK
Age	① ≤19	7.42 ± 2.70
	② 20 ~ 21	8.33 ± 2.65
	③ ≥22	8.44 ± 2.60
	*F*	7.437
	*p*	0.001
	*η^2^*	0.028
	Tukey HSD post hoc test	① < ②,③
Academic major	① Computer Science	8.01 ± 2.66
	② Civil Engineering	7.96 ± 2.93
	③ Law	8.08 ± 2.57
	④ Business Administration	7.93 ± 2.69
	*F*	0.076
	*p*	0.973
Academic year	① 1^st^ year	7.36 ± 2.60
	② 2^nd^ year	8.39 ± 2.59
	③ 3^rd^ year	8.31 ± 2.83
	*F*	8.401
	*p*	<0.001
	*η^2^*	0.032
	Tukey HSD post hoc test	① < ②,③
Residence	① Urban	8.29 ± 2.72
	② Rural	7.86 ± 2.68
	*t*	1.675
	*p*	0.095
Monthly living expenses (CNY)	① ≤1,000 CNY	7.03 ± 3.06
	② 1,001 ~ 1,500 CNY	7.93 ± 2.66
	③ 1,501 ~ 2000 CNY	8.48 ± 2.49
	④ >2000 CNY	7.86 ± 3.09
	*F*	2.878
	*p*	0.036
	*η^2^*	0.017
	Tukey HSD post hoc test	① < ③
Sexual experience	① Yes	8.97 ± 2.81
	② No	7.86 ± 2.66
	*t*	2.997
	*p*	0.004
	Cohen’s *d*	0.397

### 3.5 Distribution of HPV information channel characteristics

In terms of CT, 232 participants (44.7%) identified professional medical and health channels as their main sources of HPV information. Among all professional channels, professional medical websites or applications were the most frequently used (*n* = 160, 30.8%). The other 287 participants (55.3%) primarily accessed HPV information through non-professional channels, with social media platforms being the most common source in this group (*n* = 100, 19.3%). Detailed information is provided in Table 5.

**Table 5:** Distribution of CT among participants.

Channel category	Specific information channel	Frequency (*n*)	Percent (%)
Professional medical and health channels	Professional medical websites or applications	160	30.8
	Health education and communication from medical staff	15	2.9
	Promotional materials released by medical and health organizations	57	11.0
	Total	232	44.7
Non-professional medical and health channels	Traditional media	72	13.9
	General information websites or apps	22	4.2
	Social media platforms	100	19.3
	Communication with family, friends and classmates	93	17.9
	Other sources	0	0
	Total	287	55.3

Regarding CTR, 443 participants (85.4%) reported higher trust in professional medical and health channels. Promotional materials released by medical and health organizations were the most trusted source among all professional channels (*n* = 169, 32.6%). Only 76 participants (14.6%) reported higher trust in non-professional channels, among which communication with family, friends, and classmates was the most trusted source (*n* = 25, 4.8%). Detailed information is provided in Table 6.

**Table 6:** Distribution of CTR among study participants.

Channel category	Specific information channel	Frequency (*n*)	Percent (%)
Professional medical and health channels	Professional medical websites or applications	168	32.4
	Health education and communication from medical staff	106	20.4
	Promotional materials released by medical and health organizations	169	32.6
	Total	443	85.4
Non-professional medical and health channels	Traditional media	24	4.6
	General information websites or apps	10	1.9
	Social media platforms	17	3.3
	Communication with family, friends and classmates	25	4.8
	Other sources	0	0
	Total	76	14.6

### 3.6 Correlations among core study variables

Pearson correlation analyses were conducted to examine the bivariate associations between HIL, AISB, and HK. All three core variables showed statistically significant positive correlations with one another. Specifically, the strongest positive correlation was observed between HK and AISB (*r* = 0.486, *p* < 0.01). Detailed information is provided in Table 7.

**Table 7:** Pearson correlation coefficients for AISB, HIL, and HK (*r*-value).

Variable	AISB	HIL	HK
AISB	1	0.312^**^	0.486^**^
HIL	0.312^**^	1	0.256^**^
HK	0.486^**^	0.256^**^	1

** *p* < 0.01 (two-tailed). Values on the diagonal represent the correlation of each variable with itself.

### 3.7 Mediation effect of HIL between AISB and HK

The indirect effect of AISB on HK through HIL was tested using Model 4 of the PROCESS macro. After controlling for all covariates, AISB was positively associated with HIL (*B* = 0.795, *p* < 0.001), and HIL was positively associated with HK (*B* = 0.070, *p* < 0.001). The indirect effect of AISB on HK via HIL was statistically significant (indirect effect = 0.056, 95% bootstrap CI [0.019, 0.093]), indicating a significant partial mediating role of HIL. The direct effect of AISB on HK remained significant (*B* = 0.557, *p* < 0.001), accounting for the majority of the total effect. These results support H1, indicating that HIL partially mediates the relationship between AISB and HK (Table 8).

**Table 8:** Results of the mediation model (Model 4).

Path/effect	*B*	SE	*t*	*p*	95% CI
Path AISB → HIL	0.795	0.108	7.371	<0.001	[0.583, 1.007]
Path HIL → HK	0.070	0.020	3.540	<0.001	[0.031, 0.109]
Path AISB → HK (direct)	0.557	0.051	10.980	<0.001	[0.457, 0.656]
Path AISB → HK (total)	0.612	0.049	12.562	<0.001	[0.517, 0.708]
Indirect effect (AISB → HIL → HK)	0.056	0.019 (Boot)	–	–	[0.019, 0.093] ^a^

*N* = 519. Each model controls for age, major, grade, residence, monthly living cost, and sexual experience. Bootstrap confidence intervals based on 5,000 resamples. The completely standardized indirect effect was 0.043 (95% Bootstrap CI [0.015, 0.072]). ^a^ Bootstrap 95% CI.

### 3.8 Moderated mediation of HIL by CT on the AISB–HK relationship

The moderating role of CT was tested using Model 14 of the PROCESS macro. After controlling for all covariates, the interaction between HIL and CT on HK was significant (*B* = −0.094, *p* = 0.012). Simple slope analysis revealed that HIL was positively associated with HK among participants who primarily relied on non-professional channels (*B* = 0.112, *p* < 0.001), but not among those who relied on professional channels (*B* = 0.018, *p* = 0.521) (Figure 2). The conditional indirect effect of AISB on HK via HIL was significant only in the non-professional channel group (indirect effect = 0.089, 95% bootstrap CI [0.040, 0.143]), whereas it was not significant in the professional channel group (indirect effect = 0.015, 95% bootstrap CI [−0.031, 0.060]). The index of moderated mediation was significant (index = −0.075, 95% bootstrap CI [−0.142, −0.011]). These results support H2, indicating that CT moderates the indirect effect of AISB on HK through HIL (Table 9).

**Figure 2:**
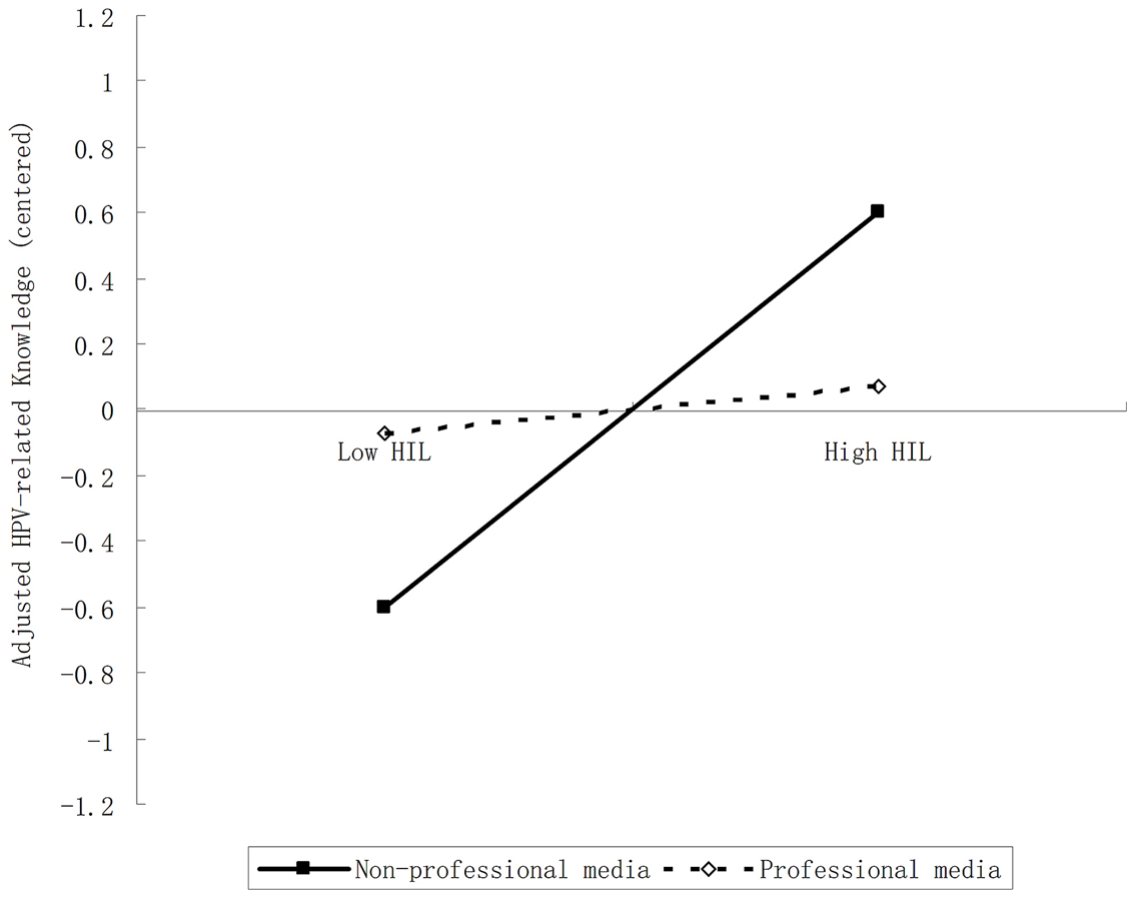
Simple slope plot illustrating the moderating effect of CT on the association between HIL and HK.

**Table 9:** Moderated mediation model with CT as moderator.

CT	Path HIL → HK			Indirect effect AISB → HIL → HK	
	*B*	95% CI	*p*	*B*	95% CI ^a^
Non-professional (0)	0.112	[0.062, 0.163]	<0.001	0.089	[0.040, 0.143]
Professional (1)	0.018	[−0.038, 0.074]	0.521	0.015	[−0.031, 0.060]
Index of moderated mediation				−0.075	[−0.142, −0.011]

Each model controls for age, major, grade, residence, monthly living cost, and sexual experience. The interaction term (HIL × CT) was significant, B = −0.094, *p* = 0.012. ^a^Bootstrap 95% CI based on 5,000 resamples.

### 3.9 Moderated mediation of HIL by CTR on the AISB–HK relationship

The moderating role of CTR was tested using Model 14 of the PROCESS macro. After controlling for all covariates, the interaction between HIL and CTR on HK was significant (*B* = −0.161, *p* = 0.005). Simple slope analysis showed that HIL was positively associated with HK in both groups, but the association was substantially stronger among participants who trusted non-professional channels (*B* = 0.210, *p* < 0.001) than among those who trusted professional channels (*B* = 0.049, *p* = 0.019) (Figure 3). The conditional indirect effect of AISB on HK via HIL was significant in both groups (non-professional trust group: indirect effect = 0.167, 95% bootstrap CI [0.086, 0.268]; professional trust group: indirect effect = 0.039, 95% bootstrap CI [0.001, 0.078]). The index of moderated mediation was significant (index = −0.128, 95% bootstrap CI [−0.235, −0.046]). These results support H3, indicating that CTR moderates the indirect effect of AISB on HK through HIL (Table 10).

**Figure 3:**
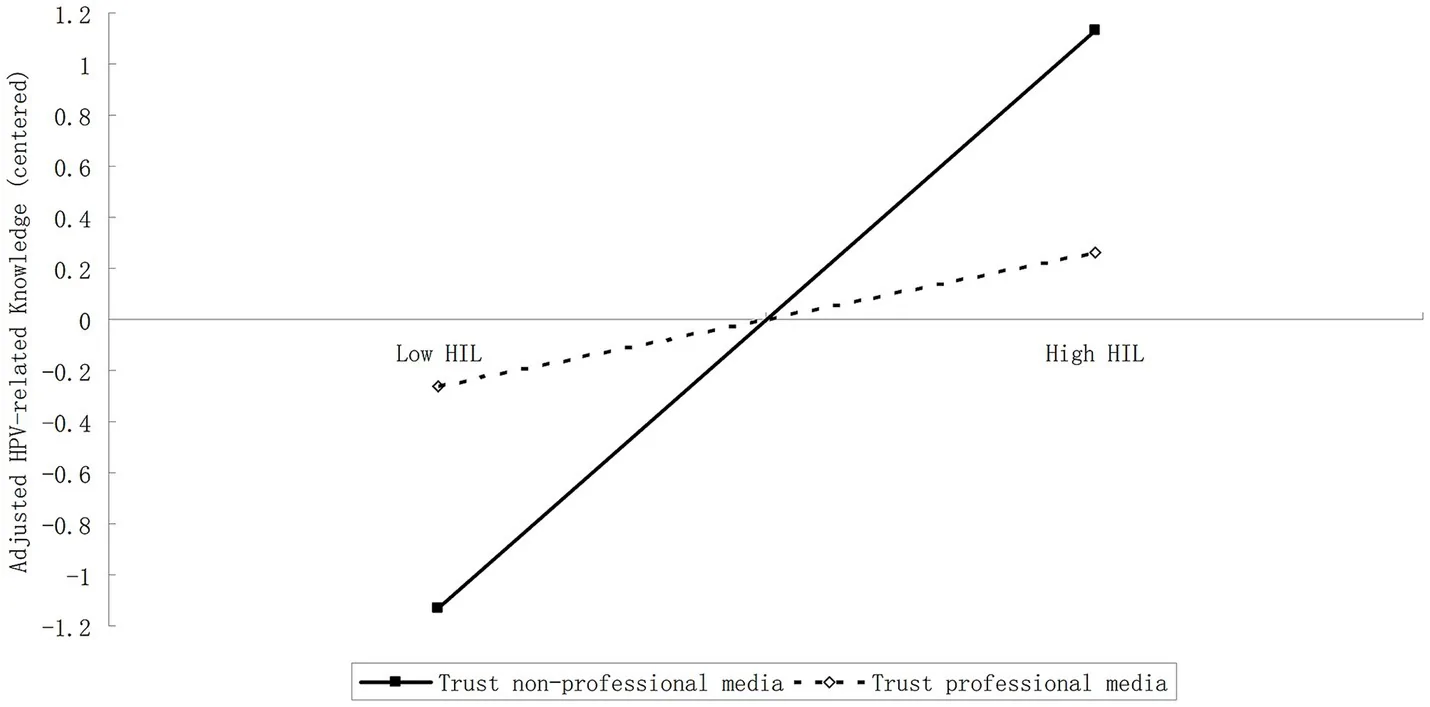
Simple slope plot illustrating the moderating effect of CTR on the association between HIL and HK.

**Table 10:** Moderated mediation model with CTR as moderator.

CTR	Path HIL → HK			Indirect effect AISB → HIL → HK	
	*B*	95% CI	*p*	*B*	95% CI ^a^
Non-professional trust(0)	0.210	[0.106, 0.314]	<0.001	0.167	[0.086, 0.268]
Professional trust (1)	0.049	[0.008, 0.090]	0.019	0.039	[0.001, 0.078]
Index of moderated mediation				−0.128	[−0.235, −0.046]

Each model controls for age, major, grade, residence, monthly living cost, and sexual experience. The interaction term (HIL × CTR) was significant, B = −0.161, *p* = 0.005. ^a^Bootstrap 95% CI based on 5,000 resamples.

## 4 Discussion

This study examined the relationships among AISB, HIL, and HK among female undergraduates, and further investigated whether these relationships were contingent upon information channel characteristics. Three main findings emerged. First, HIL partially mediated the positive relationship between AISB and HK, suggesting that active seeking behavior contributes to knowledge acquisition both directly and through the enhancement of HIL. Second, channel type moderated the HIL–HK pathway: HIL was significantly associated with HK only among participants who primarily relied on non-professional channels, but not among those using professional channels. Third, channel trust preference also moderated the HIL–HK pathway; although HIL was significantly associated with HK regardless of trust orientation, the magnitude of this association was approximately four times stronger among those who trusted non-professional channels. Collectively, these findings point to a consistent pattern: the value of health information literacy in facilitating HK is amplified in less reliable information environments—whether defined by the objective nature of the channel or the subjective trust placed in it. The following sections discuss the implications of these findings in detail.

### 4.1 Demographic differences in HK, HIL, and AISB

Several demographic characteristics were associated with the core study variables, with monthly living expenses emerging as the most consistent correlate across all three outcomes.

Monthly living expenses were significantly associated with AISB, HIL, and HK. Post-hoc tests revealed that participants with higher monthly living expenses tended to score higher on AISB, HIL, and HK than those with lower expenses, although the specific pattern of pairwise differences varied across the three measures. This finding is consistent with the conclusion drawn in numerous previous studies that economic inequality leads to health inequality (42–44). At a more granular level, a substantial body of research has also demonstrated that personal economic status is positively associated with health information literacy (45–47), health information-seeking behavior (47–49), and health-related knowledge (50–52).

Age and academic year were significantly associated with HK but not with AISB or HIL. The results indicated that knowledge levels tended to be higher among older students or those in more advanced academic years. Sexual experience was also significantly associated with HK but not with AISB or HIL. Participants who reported having sexual experience scored higher than those without such experience. These findings align with prior studies (53–57). Given that these findings have been extensively reported and discussed in previous literature, further analysis is not warranted here.

### 4.2 The dual pathways from AISB to HK: the mediating role of HIL

The present study found that HIL partially mediated the relationship between AISB and HK, supporting H1. This finding reveals a dual-pathway mechanism: AISB contributes to HK both directly and indirectly by forging HIL, which then facilitates knowledge internalization.

#### 4.2.1 The indirect pathway: a “practice–empowerment–internalization” model

The observed indirect effect is consistent with a sequential association pattern: higher AISB was associated with higher HIL, and higher HIL, in turn, was associated with greater HK. We interpret this pattern through a conceptual framework termed “practice–empowerment–internalization.” However, given the cross-sectional design, this framework should be understood as a theoretical interpretation of the observed statistical associations rather than as evidence of a causal sequence or temporal ordering.

The first stage of this model posits that active seeking may function as a “training ground” for health information literacy. While extensive prior research has established information literacy as an antecedent to seeking behavior (58–60), our findings suggest the possibility of a reverse pathway: that active seeking was associated with higher literacy. This can be understood through Lloyd’s (61) conceptualization of information literacy as a socio-cultural practice. According to Lloyd, literacy is not merely a set of decontextualized skills but is enacted and shaped within specific information landscapes. In the context of HPV, our descriptive data illustrate a landscape marked by a striking tension: although 55.3% of participants primarily used non-professional channels to access HPV information, 85.4% expressed trust in professional medical channels. This discrepancy may prompt individuals to constantly verify, compare, and evaluate across conflicting channels—practices that involve intensive exercises in information appraisal and integration, the core components of HIL. Empirically, Asemi et al. (62) similarly demonstrated that a positive attitude toward information seeking increases the likelihood of information literacy competence. Additionally, this finding also resonates with Limberg and Sundin’s (63) assertion that information seeking and information literacy are “two sides of the same coin”.

In the second stage, higher health information literacy was associated with a greater tendency to integrate information into knowledge. Higher HIL was associated with moving beyond rote memorization of isolated facts toward a more integrated understanding. As described by Bruce (64), “synthesizing information to construct knowledge” represents a higher-order manifestation of information literacy. In our study, although the knowledge measure largely assessed basic facts, certain items required modest information integration. For instance, correctly answering when HPV vaccination is most effective demands connecting the concepts of sexual transmission and pre-exposure prophylaxis, rather than merely recalling the recommended age. Participants with higher HIL were more likely to synthesize fragmented information and form such coherent cognitive links. Prior studies have documented positive associations between health literacy and disease-specific knowledge (23–25, 30–32); our study extends this line of inquiry by providing evidence consistent with a specific role of health information literacy in this process.

Taken together, this indirect pathway is consistent with the “practice–empowerment–internalization” framework: higher AISB was associated with stronger HIL, which in turn was associated with greater HK.

#### 4.2.2 The direct pathway: consistent with prior evidence, with unexplained mechanisms

Beyond the indirect route, the significant direct effect of AISB on HK indicates that active seeking was also directly associated with knowledge, beyond the indirect pathway through HIL in our model. This finding is consistent with a robust body of literature showing that health information seeking is a direct predictor of knowledge across various contexts (17–21). Such direct gains likely represent factual information that can be acquired through simple recognition and memory—such as the number of vaccine doses required—without necessitating higher-order evaluation or synthesis.

However, it is important to acknowledge that this direct effect may encompass additional indirect mechanisms not captured in the current model, as other potential mediators were not included. Future research employing multiple-mediator models is warranted to disentangle these alternative pathways.

### 4.3 Moderating effects of information channel characteristics

The present study found that both CT and CTR significantly moderated the indirect effect of AISB on HK through HIL, supporting H2 and H3. These two moderating effects converge on a unified pattern: the positive association between HIL and HK was stronger in less reliable information environments.

#### 4.3.1 Moderating effect of CT on the pathway from HIL to HK

The results revealed that the positive association between HIL and HK was significant only among participants who primarily relied on non-professional channels, whereas this relationship was not significant among those who used professional channels. This finding indicates that the association between HIL and HK was contingent upon the objective quality of the information environment.

This pattern may be explained by the differential information quality across channel types. Non-professional channels (e.g., social media—the most commonly used sources in this group) feature highly heterogeneous information quality, with evidence-based scientific content coexisting with misleading claims, anecdotal reports, and health misinformation (14, 65, 66). In such unregulated information environments, the association between HIL and HK was stronger, possibly reflecting the greater demand for information evaluation in these settings; accordingly, the HIL–HK association was most pronounced in low-quality information settings. In contrast, information from professional medical and health channels undergoes rigorous expert review and institutional quality control, ensuring high and homogeneous accuracy and authority. Within this, in principle, standardized information environment, individuals across different literacy levels are exposed to largely consistent, reliable core knowledge, which may account for the weaker HIL–HK association observed in this group.

In summary, the moderating effect of CT highlights the context-dependent nature of the HIL–HK association. The association was most evident in non-professional information environments.

#### 4.3.2 Moderating effect of CTR on the pathway from HIL to HK

The results revealed that CTR also significantly moderated the HIL–HK relationship. HIL was positively associated with HK in both trust groups, but the magnitude of the positive association in the non-professional trust group was approximately four times that in the professional trust group, and the conditional indirect effect showed a proportional difference. These findings indicate that CTR, unlike CT, was associated with the magnitude of the HIL–HK association rather than its statistical significance.

This divergent pattern may be interpreted in light of the gap between trust and exposure. Trusting professional medical channels does not guarantee that one’s actual information environment is exclusively populated by professional content. In the contemporary digital landscape, health information reaches individuals through multiple intersecting channels, many of which operate outside the boundaries of conscious selection. In such mixed information environments, the HIL–HK association may reflect the need to distinguish between trusted professional content and casual exposure to non-professional content.

Beyond this structural gap, cognitive factors may further shape the moderating pattern of channel trust. Many theories of cognitive consistency converge on the assumption that information inconsistent with one’s beliefs tends to elicit negative feelings (67) and prompt closer scrutiny (68), whereas information consistent with one’s beliefs tends to be processed more fluently and is more likely to be accepted as true (69, 70). Participants who trust professional channels may experience cognitive disfluency when encountering non-professional content that contradicts established medical knowledge. This disfluency may serve as an implicit warning signal that is associated with more careful evaluation. Conversely, participants who trust non-professional channels may process information from these sources with greater fluency and familiarity, which may be associated with reduced natural skepticism. For these individuals, the association between HIL and HK was most pronounced.

In summary, the moderating effect of CTR is consistent with the possibility that trust is associated with how individuals process the health information they encounter. When trust is misplaced, the absence of cognitive skepticism may be associated with a stronger HIL–HK association.

#### 4.3.3 Comparative analysis of two moderating effects

Taken together, the moderating effects of CT and CTR reveal a consistent yet differentiated picture. Both moderators significantly conditioned the HIL–HK pathway and converged on the same core pattern: the positive association between HIL and HK was stronger in less reliable information environments. Yet the two moderators were associated with notably different patterns.

The most salient divergence lies in the pattern of conditional effects. CT produced a qualitative distinction: the HIL–HK association was significant only in the non-professional channel group. CTR produced a quantitative distinction: the association was significant in both groups, with the magnitude differing approximately fourfold. This contrast points to a fundamental difference. Objective channel quality, when uniformly reliable, the HIL–HK association was weaker. Subjective trust, by contrast, was associated with a reduced but still significant HIL–HK association, possibly because trust does not fully shield individuals from exposure to conflicting information.

This divergence can be further interpreted through the distinction between environmental compensation and cognitive moderation. CT is consistent with the former: professional channels, by ensuring baseline information quality, may reduce the observed association between HIL and HK. CTR is consistent with the latter: trust may be associated with cognitive processing, such that epistemic vigilance varies with the trust orientation. The former is an external factor rooted in the information environment; the latter is an internal factor rooted in cognitive processes.

Despite these differences, the two moderating effects converge on a unified theoretical proposition. Whether defined objectively by CT or subjectively by CTR, the reliability of the information environment was associated with the strength of the HIL–HK association.

### 4.4 Implications for public health practice

The findings of this study carry several implications for public health practice, particularly in the context of HPV-related health education and vaccination promotion among university students.

First, address the mismatch between daily information exposure and credibility. Medical and health institutions should expand their presence on youth-focused social platforms to deliver authoritative HPV education content, while platforms should strengthen health content review to help reduce misinformation (14, 71), narrowing the accessibility–credibility gap.

Second, embed HIL training in college reproductive health curricula. Targeted interventions should prioritize first-year students and those with lower living expenses, with the aim of enhancing their information evaluation skills and potentially reducing HPV knowledge disparities.

Third, adopt stratified, targeted intervention strategies. Basic HPV knowledge promotion should focus on younger and economically disadvantaged students, while vaccination and screening education should target senior students and those with sexual experience to better match content to their needs.

Fourth, develop peer education programs to extend the reach of evidence-based HPV information. Students with solid HK, adequate HIL, and strong communication skills can be recruited as peer educators. Leveraging shared identity and daily peer interaction, this model may improves the acceptability and penetration of health education on campus (72, 73).

Fifth, establish multi-stakeholder collaboration. University hospitals, student affairs departments, and local center for disease control and prevention (CDC) should coordinate to integrate offline professional lectures and online popular science content, leveraging the complementary strengths of professional and non-professional channels.

### 4.5 Limitations and future directions

Several limitations of this study should be acknowledged. First, the cross-sectional design precludes causal inference. Although the mediation model tested in this study is grounded in theoretical assumptions, the temporal ordering of AISB, HIL, and HK cannot be empirically established. Accordingly, all reported associations—including the indirect effects—should be interpreted as cross-sectional and statistical in nature, consistent with but not proving the proposed model. Longitudinal or experimental designs are needed to verify the proposed pathways.

Second, the measurement of AISB, although achieving acceptable internal consistency (Cronbach’s *α* = 0.80), relied on a brief three-item scale developed for this study that has not undergone extensive psychometric validation. Future research should consider employing validated multi-item instruments to capture additional facets of health information seeking behavior.

Third, the internal consistency of the EHIL scale was marginal in this sample (Cronbach’s *α* = 0.67). Although marginal internal consistency may yield conservative estimates of effect sizes, significant associations were still detected. Future studies using this instrument in comparable populations should consider item refinement to improve measurement precision.

Fourth, the sample was drawn from a single comprehensive university and consisted exclusively of female undergraduates. Future research should replicate these findings in more diverse populations, including male students, non-university youth, and multi-center samples across different regions and institution types.

Fifth, all measures were based on self-report, which is subject to social desirability bias and recall inaccuracies. Future studies could strengthen the validity of these findings by incorporating objective measures or direct observation of information seeking behavior.

Sixth, the operationalization of CT and CTR as binary variables captured broad distinctions but inevitably simplified the complexity of individuals’ information environments. Future studies could adopt person-centered approaches, such as latent profile analysis, to identify distinct configurations of information channel use and trust, providing a more nuanced understanding of how channel characteristics moderate the literacy–knowledge relationship.

Finally, because class identifiers were not recorded, we were unable to formally account for clustering by class. Although academic major and academic year—the stratification variables—were included as covariates and represent the primary sources of between-class homogeneity, future studies should retain class-level identifiers to enable formal adjustment for clustering effects.

## 5 Conclusion

This study examined the relationships among AISB, HIL, and HK among female undergraduates, and further investigated the moderating roles of information channel characteristics. The findings revealed that HIL partially accounted for the association between AISB and HK, consistent with a dual-pathway pattern in which AISB was associated with HK both directly and indirectly through HIL. More importantly, both CT and CTR moderated this indirect pathway, converging on a unified pattern: the positive association between HIL and HK was stronger in less reliable information environments. When the information environment is characterized by professional quality assurance, higher channel reliability was associated with a weaker HIL–HK association. When such assurance was absent or trust was misplaced, the HIL–HK association was most pronounced. These findings underscore the need for integrated public health strategies that simultaneously strengthen professional information environments and equip individuals with the skills to navigate increasingly complex health information landscapes.

## Data Availability

The raw data supporting the conclusions of this article will be made available by the authors, without undue reservation.

## Footnotes

1
https://www.wjx.cn/
